# Exophthalmic Goitre; Its Etiology, Symptoms and Treatment1Read by invitation before the Buffalo Academy of Medicine, October 14, 1901.

**Published:** 1902-01

**Authors:** Casey A. Wood

**Affiliations:** Chicago, Ills., Professor of Clinical Ophthalmology in the University of Illinois.


### BUFFALO MEDICAL JOURNAL.

VOL. XLI.—LVII. JANUARY, 1902. NO. 6.

### ORIGINAL COMMUNICATIONS.

### Exophthalmic Goitre; its Etiology, Symptoms and Treatment.[1]

BY CASEY A. WOOD, M. D., Chicago, Ills.,
Professor of Clinical Ophthalmology in the University of Illinois.

I HAVE employed the term exophthalmic goitre because I do not believe that one can use the expression, "Graves's" or "Basedow's disease," without doing injustice to the memory of an Englishman, C. H. Parry, who intelligently observed and described this symptom-complex in 1786, although his writings were not published until 1825, some fifteen years before Basedow's first article appeared in Casper's *Wochenschrift für die gesammte Heilkunde.* The publication of Parry's observations also antedates Graves's description of the disease contained in his famous lectures contributed to the London *Medical and Surgical Journal* for 1835. So far as priority of publication is concerned, probably both Basedow and Graves are put out of court by the superior claims of the Italian, Giuseppe Flajani. In his *Collezione d' osservazioni e riflessioni di Chirurgia*, published in four volumes at Rome in 1802, a fairly accurate account is given of an example of this curious affection,—although we do not know that he appreciated the correct symptoms of the disease.

In some Italian works, also, reference is made to *il morbo di Flajani*, although in recent years the term *gozzo esoftalmico* or *il morbo di Basedow* is employed. I would refer those interested in the matter to the article on *Goitre Exophthalmique* in Dechambre's *Dictionnaire Encyclopédique*, and to the introduction to Möbius' *Die Basedow'sche Krankheit.* In any event, and for obvious reasons, the expression "exophthalmic goitre" is to be preferred until, at least, we can agree upon or

1. Read by invitation before the Buffalo Academy of Medicine, October 14, 1901.

have learned more than we now know about the pathology of this interesting array of signs and symptoms.

That exophthalmic goitre is the result of a systemic poisoning, produced by deranged function of the thyroid gland, there can be but little doubt. The chief difficulties in the way of a satisfactory study of the disease arise, when we endeavor to explain just how such functional derangement occurs and what is the character of the leucomaines, or the products that directly bring about the toxic symptoms. The discussion of the pathology of this form of goitre, involving as it does the whole question of toxemia, as well as the probable function of the thyroid, is one of peculiar interest, but time will hardly permit of it this evening; suffice it to say, that notwithstanding the defects in the "hyper-" and "dysthyroidation" theory it is the one that to the present time explains most of the facts. Moreover, it is well known that the administration of thyroid extract increases the violence of many of the symptoms and that, speaking generally, such treatment as reduces the mass of the enlarged gland, or the volume of its secretion, exerts a favorable influence on the other manifestations of the disease. Notthaft and others have reported cases in which overdoses of thyroidin brought on cardiac palpitation, marked sweating, difficult breathing, exophthalmus, fast pulse, tremor, as well as most of the eye signs of this affection. When we also recollect that myxedema, the clinical antipodes of exophthalmic goitre, is associated with atrophy and lost function of the thyroid, only one conclusion seems possible.

It may well be objected to the foregoing statement that no light is thrown upon the cause of this depraved action of the thyroid, and we are constantly obliged to consider those hypotheses that represent the disease as essentially a lesion of the cervical sympathetic,—a view of the case that, once rejected, has lately been revived in the treatment of exophthalmic goitre by the removal of the cervical ganglia. It was, as you know, at one time held that strumous or other lesion of the great sympathetic and subsequent paralysis of its vasomotor nerves are competent to produce the goitre and the exophthalmus, together with the dilatation and pulsation of the carotid and other arteries. At the same time this alteration is expected to account for the tachycardia, by permanent irritation of the cardiac excito-motor nerves that also run to the cervical sympathetic. Such a double-headed hypothesis, whose two chief factors seem mutually exclusive, has never, so far as I know, been elevated to the dignity of a theory either by evidence derived from experiments on the lower animals or by clinical observations of patients where paralysis of the nerve has occurred. Again, the pupillary reactions in Graves's disease are usually normal when they would inevitably be disturbed in disease of the sympathetic.

These difficulties led Prof. Sattler (then of Erlangen now of Leipzig) to advance a theory that to some extent removes them. He postulates a lesion of that portion of the central vasomotor neuron that presides over the vasomotor nerves of the thyroid, and of the post-ocular tissues within the orbit as well as an alteration in the cardio-inhibitory center of the pneumogastric. In further support of Sattler's theory, Fitzgerald in 1883 pointed out that many cases of exophthalmic goitre are complicated with symptoms clearly cerebral, as for instance, glycosuria, paresis of the external recti muscles and paralysis of the associated movements of the globe. The chief objection, to my mind, that resides in the Sattler theory is that beyond Filehne's experiments on the restiform bodies (when division of these produced impaired function of the vagus, exophthalmus and vasomotor paresis in the ears, thyroid and anterior part of the neck) there are no post mortem findings to support it. Furthermore, it is necessary to suppose several circumscribed, isolated, invariable and unrelated lesions which are without our experience in practical pathology.

The statement that acute iodism and exophthalmic goitre are practically the same condition has received the sanction of several observers. Jaunin and G. Gautier, both writing in the *Rêvue médicale de la Suisse Romaine* for 1899, describe cases in which the exhibition of iodine in considerable doses brought on all the symptoms of Graves's disease, except the marked proptosis.

The *exciting causes*, also, can have only a passing mention. Of some eleven cases observed, more or less closely by me, two only were in men,—one of twenty-four, the other twenty-two years of age. All the others were women of from nineteen to thirty-three years old. Graves' disease is in that sense a "female" affection. There can be no doubt but that anemia and chlorosis are often present. Overwork, worry, fright, catching cold (thereby bringing on an acute or chronic thyroiditis) great mental excitement, excessive venery, insomnia, as well as many other causes have been assigned by both physician and patient for the diseased manifestations. Owing to the preference of exophthalmic goitre for women it has been supposed, in some way or other, to depend on alterations in function or organic changes in one or other of the reproductive organs, just as its connection with nasal disease, with the so-called "strumous" condition, with floating kidney, disease of the bladder, etc., has been noted.

Apart from the eye symptoms let me remind you of the hypertrophy and degeneration of the thyroid gland, of the palpitation of the heart, (usually the first of the cardinal symptoms to appear and the most constant of them all), its irregularity, its occasional enlargement, the occurrence of systolic murmurs of the anemic type, the pulsation of the enlarged arteries,—particularly in the carotids in the neck and the abdominal aorta (occurring with the tachycardial), the weak and soft radial pulse and the pulsation and enlargement of the spleen, noticed by C. Gerhardt. As a continuation of this catalogue I may mention the extreme nervousness, often bordering on and sometimes complicated with hysteria, the fine tremor or tremulousness, the insomnia, the vertigo, the paresthesiæ, as well as the muscular cramps and the various pareses. Other skin symptoms are (Vigoroux's sign) the diminished electric skin resistance, the various dermal eruptions and the sweating,—the last often alternating with extreme dryness.

I may perhaps be allowed here to speak of a symptom that I have noticed, once only, in a patient with exophthalmic goitre, whom I saw some fifteen years ago. I refer to a peculiar form of fetid night sweats. I spoke of it at that time to Prof. Sattler because I had been unable to find any mention of it in the literature then at my command. The patient, one of the two men whom I have seen with the disease, had attacks of the most foul smelling diaphoresis I have ever experienced and they persisted as long as I attended him, requiring disinfectant sponge baths for their relief. Sattler had not seen such a case; you may imagine my satisfaction, therefore at finding several years afterwards that Basedow himself mentions an example of it.

Next in order comes swelling of other glands besides the thyroid lymph glands, the spleen, the thymus, often appearing with tumefaction and other affections of the joints. We also notice the dry cough, the weakness of the voice, the difficulty of breathing and the occurrence of Bryson's sign (defective expansion of the chest in respiration), disorders of digestion with emaciation and micturition (with glycosuria), so well described by Charcot. Probably the diarrhea, vomiting, urination, and the like, are the direct efforts of nature to get rid of the thyroid poison.

I would not have you suppose that in claiming much of value for the eye symptoms, I am impelled to do so mainly because of my studies as an ophthalmologist. I think you will agree with me that the profession has not yet recovered from the impression early given it, that ocular symptoms are matters of deep mystery not to be penetrated except in case of the direst need. As a matter of fact the ophthalmic manifestations of Basedow's disease are valuable because of their easy detection and because of the fact that one or more of them invariably occur (usually early) during the course of the disease, and because in doubtful cases they may be relied upon to differentiate exophthalmic goitre from other forms of exophthalmus, tremor, tachycardia, struma, and the like.

The *exophthalmus* is commonly the first eye symptom to attract the attention of the observer. It gradually increases until the lids may be unable to close over the eyeball, thus rendering the globe liable to infection and interfering with the proper drainage of the conjunctival sac. In the early stages the ball may be pushed, without producing pain, back into the orbit but later this cannot be done. This fact can be explained if we remember the direct cause of the proptosis. At first the bulging is produced by the enlargement of the blood-vessels only and possibly the filling of the lymph channels. Later, a permanent pad of connective tissue and fat is deposited. When a cure is effected in a chronic example of the disease, the other symptoms usually disappear before the exophthalmus,—probably because the orbital fat does not readily undergo absorption. Occasionally, one eye subsides more quickly than the other. This sign can almost always be differentiated from orbital aneurism and tumor, by the fact of the proptosis in goitre occurring directly forward and of its being bilateral. We must not forget, however, that there is such a thing as unilateral exophthalmus in genuine Graves's disease. As Fitzgerald pointed out, it is usually the right eye and the right side of the body that are effected in these monolateral cases.

Before the exophthalmus appears *retraction of the upper lid and widening of the interpalpebral fissure* (Dalrymple's sign) is commonly seen. It is one of the earliest and most regular symptoms of the disease. Our forefathers regarded it as a result of the exophthalmus. We know, on the contrary, that the proptosis seems more pronounced on account of the wide open lids. Continual palpebral retraction to an extent that exposes the white sclera between lid edges and cornea, above and below, is practically always a sign of disease. It is almost invariably a symptom of Basedow's disease, although the "staring eye" or "glotzauge," as our German friends call it, is occasionally seen in certain forms of mania, in tetanus, in hysteria and other affections.

*Infrequent winking* is also an important and constant sign of exophthalmic goitre. This is known as Stellwag's sign, although most authorities persist in confounding or including with it the sign of Dalrymple. The truth is, so far as I have been able to trace it, that in 1869, Stellwag first described the sign that bears his name and drew attention to the retraction of the lids as the most noticeable cause of the glotzauge. But Dalrymple, had years before (*London Lancet*, May 26, 1849,) published a definite description of this same phenomenon.

*Graefe's sign* is a most valuable one. It occurs early, is quite constant, and although it may be present in Thomsen's disease and sometimes fails altogether, it should always be searched for in doubtful cases. It consists in an impairment of the consensual movements of the upper lid in association with the eyeball. In a normal eye, when the globe is rotated downward, the upper lid-edge follows it, covers a portion of the upper cornea and preserves about the same distance from the sclero-corneal junction throughout the whole excursion. Much of this diagnostic value, which is great, depends its mode of application. It is better to cover one eye and ask the patient, with his head in the primary position and at ease, to fix the point of a pencil held eighteen inches from his face and on a level with the forehead. The pencil end is now slowly lowered, the head all the while in the primary position, and the pencil kept eighteen inches from the body, until it reaches a point opposite the ensiform cartilage. In exophthalmic goitre the upper lid, during this excursion, will be noticed to lag behind and even to expose a zone of white sclera between it and the cornea.

*Insufficiency of convergence*, or the sign of Möbius, depends upon the fact that the stretched internal recti muscles are unable to move the bulging eyes inward to the same extent and with the same facility that they can normally situated globes. It is necessary for binocular vision that the visual axes should be directed at about the same angle toward the object to be fixed. Möbius claims that in Basedow's disease the excursions of the eyes in other directions are practically normal but that, without producing double vision, weakness of convergence is a characteristic of the majority of cases. It must not be forgotten that symmetrical paresis of the external recti muscles is sometimes a sign of exophthalmic goitre, and then we may have apparent excess of convergence.

Cases are on record, although it has never been my fortune to observe an example of this lesion, in which a single muscle was paralyzed while others have published instances of complete ophthalmoplegia externa. These are probably, in most cases, the result of nuclear disease.

What I regard as most valuable evidence of the disease is *Becker's sign*, first described by Otto Becker in the *Wiener klin. Wochenschrift* for 1873. I refer to spontaneous pulsation of the retinal arteries. This sign is, of course, met with in other conditions,—glaucoma, aortic regurgitation, and the like,—but it is an aid to diagnosis that we cannot afford to ignore. The pulsation does not, as a rule, extend beyond the optic disc (as in most cardiac lesions) and it is usually accompanied by tortuous and enlarged veins.

*Epiphora*,—watery eyes,—has been seen a number of times and may be very troublesome. I observed it in one of my cases—a woman—and Emile Berger mentions it as the earliest of the ocular symptoms in two of the histories reported by him. He speaks of it as a nervous affection of the lachrymal gland, but in the case under my own observation it seemed to result from the excessive proptosis, causing displacement of the puncta lachrymalia.

*Dryness of the eyes* is one of the commonest complaints made by patients. It is probably due to the absence of winking (the process by which chiefly the cornea and the bulbar conjunctiva are cleansed in the normal eye) and the exposure of the wide open eyes to wind, dust, smoke and other irritants.

One wonders that traumatic and other forms of keratitis, as well as infective ulcer of the cornea do not more frequently occur in the partially protected eyes of this disease. Basedow has himself drawn our attention to its occasional occurrence and gives an account of a merchant, aged 50, who lost both eyes from perforating ulcer, apparently induced by driving in an open carriage along dusty roads and with his eyes much exposed to the wind.

*Loss of sensation of the cornea* and conjunctiva has several times been noticed and doubtless has been overlooked in many other cases, as, of course, it is an objective symptom and does not attract the attention of the patient. A very pretty theory might be built upon the fact of its presence to account for at least some of the eye symptoms. I give it to you for what it is worth. As is well known, the incentive to normal winking and consequent cleansing of the eyeball is chiefly dryness of the anterior segment of the globe due to evaporation from the exposed surface. To this is added the foreign body sensations set up by fine dust and other particles. In the same way the lid maintains the same relative position upon the globe in all positions of the latter, in virtue of the orientation made possible by the same lid-eyeball-sense. Indeed, the maintenance of a certain space between the lid edges,—the usual size of the interpalpebral aperture,—depends largely upon the fibres of the fifth nerve supplied to all the parts. In exophthalmic goitre this sensibility is destroyed, hence the eye signs. Probability is lent to this hypothesis when we bring about this asensitive condition artificially. A few drops of a 4 per cent. solution of cocaine instilled into each eye at two minute intervals will, in from ten to twenty minutes, enable one to induce and study the Graefe, Dalrymple, and Stellwag symptoms without difficulty. Even the appearance of the proptosis is sometimes induced by the fixed stare that a cocainised eye not infrequently assumes.

Circumscribed edema of the eye-lids, as well as pigmentation of their skin surface, has been occasionally seen, but these are probably mere accidents and form a part of the dermal changes seen elsewhere on the body.

It seems remarkable that so far no definite blood changes have been discovered in this form of toxemia. Doubtless in the future we shall owe much in diagnosis to the careful analysis of those highly complex organic blood compounds almost certainly present in goitre patients, about whose origin and meaning we now know so little.

It is not my purpose to dwell upon the varieties of the disease or its complications,—hysteria, myxedema, and the like. It is well, however, to remember that to a previously existing simple goitre of the strumous variety, may be added the proptosis and the other signs of Graves's disease; that in its fullest manifestations it may develop very suddenly as an acute disease, the symptoms be very severe and the end may be in death. On the other hand, its usual characters are those of a slowly progressive affection tending on the whole, as Hulke has pointed out, to recovery if the patient does not die of cardiac paresis or some other intercurrent disease. Exophthalmic goitre may, as we know, exist without at least two of its three cardinal signs. One side alone may be affected by the proptosis and the other eye signs, or quite a few symptoms may be wanting, Vigouroux's, Möbius', Bryson's, and others.

It was not my intention to say much about *treatment* because I have had no experience of the operative procedures recently advised, complete and partial thyroidectomy, sympathectomy and injections into the substance of the thyroid. We have the testimony, especially of Kocher and Stokes, in favor of the first named. All things considered removal of one or more cervical ganglia seems to be the most desirable of the operative procedures, as prescribed by Jonnesco and his following. Intraglandular medication is, of course, not new except as to the medicaments employed. The last I have heard of is the injection of bile.

It is always to be remembered that medical treatment alone has sufficed to cure the disease. Brilliant results have been obtained by such different remedies that one is inclined to believe in the self-limitation of a certain class of goitres. They tend to get well, if left alone long enough. Electricity, mineral waters, hydrotherapy, large doses of sodic salicylate, belladonna, digitalis, ergotin, amyl nitrite, I have myself prescribed at various times and I think all with some benefit. I would strongly suggest that these patients live as quiet a life as possible, that they have plain, non-stimulating food and that they be induced to take as much sleep as possible. The difficulties encountered in treating severe cases of Basedow's disease are exemplified in a recent article by Tornatola (*Archivio di Ottalmologia,* May-June, 1901.) He was called upon to prescribe for a woman with fully developed Graves's disease in which the exophthalmus was very pronounced, 1.5 cm. in the right and 1 cm. in the left eye. An ulcer occupied the two lower quadrants and two-thirds of the thickness of the right cornea. The lids covered only about one-third of the globe. In the left eye there was a smaller ulcer in the center of the lower quadrant. Corneas retained their sensitiveness. After the usual disinfection the lids were sutured, in such a way that the knots could be readily untied for appropriate treatment. In eight days the left ulcer healed, the right one in fifteen days. A month later she returned with a second corneal infection in both eyes, to be again cured. A third time the patient appeared with spreading ulcer in either eye, but on this occasion the lids could not be sutured over the globes, chiefly because the scarred skin of the lids no longer retained the threads and knots. Tarsorrhaphy was done but the adhesions soon gave way. Sympathectomy was then performed by Prof. Solomoni with considerable relief to the exophthalmus and the goitre; the tachycardia remained. Meantime the corneal ulceration, infiltration and conjunctival chemosis increased until the left eye had to be exenterated. Tornatola now endeavored to preserve some vision in the remaining eye and succeeded by supplementing a second tarsorrhaphy with large skin grafts from the nose and temple, until the interpalpebral aperture was finally closed. The patient's condition a year after was fairly satisfactory. Photographs and measurements show a reduction of the exophthalmus about one-half; the thyroid is "slightlv reduced" and the general health decidedly better.

On the whole J. C. Da Costa's advice with regard to excision of the thyroid is most seasonable: "If medical treatment fails and the symptoms are urgent consider the advisability of partial thyroidectomy unless the goitre is very large or extremely small, or the patient is very hysterical. Removal of a very large goitre is extremely dangerous and will not bring about cure. If the thyroid gland is but little enlarged it is not necessary to remove it. In severe hysteria the operation is often useless. Never promise a cure and always set forth the danger of the operation."

103 Adams Street.

